# Chordoid glioma in the thalamus of a child: Rare location and atypical imaging findings

**DOI:** 10.1259/bjrcr.20200108

**Published:** 2021-01-08

**Authors:** Cong Huang, Dengwei Gan, Botao Huang, Junde Luo, Xingshun Zhou, Wensheng Wang, Yujun Wang

**Affiliations:** 1 Department of Radiology, No. 926 Hospital, Joint Logistics Support Force of PLA, Kaiyuan, Yunnan, China; 2 Department of Radiology, The Affiliated Dongguan Houjie Hospital of Guangdong Medical University, Dongguan, Guangzhou, China; 3 Department of Imaging Center, Guangdong 999 Brain Hospital, Guangzhou, Guang Dong, China; 4 Department of Radiology, Tongde Hospital of Zhejiang Province, Hangzhou, Zhejiang, China

## Abstract

Chordoid glioma is a rare intracranial tumour, which usually occurs in middle-aged female patients, mainly in the third ventricle, hypothalamus and suprasellar region. It can reportedly occur in the temporal–parietal lobe, occipital horn of the lateral ventricle and left thalamus. Here, we report a case of chordoid glioma in the thalamic region of a female child, which is different from the previously reported chordoid glioma in the left thalamus. Given its atypical location and imaging findings, it is often misdiagnosed as low-grade glioma before operation. Through the study of this case, we recognized the atypical imaging manifestations of chordoid glioma in a rare location.

## Introduction

Chordoid glioma is a rare intracranial tumour. It was first proposed and named by Brat et al. in 1988. In 2016, it was listed under “other types of gliomas”, class II, in the latest World Health Organization (WHO) classification of tumours of the central nervous system.^1^ The origin of chordoid glioma is unknown, and it usually occurs in the anterior part of the third ventricle, especially in the anterior hypothalamus, but it has been reported to occur in the temporal–parietal lobe,^2^ the occipital horn of the lateral ventricle,^3^ and the left thalamus.^4^ Chordoid glioma mostly occurs in middle-aged patients aged 30–60 years, with a male-to-female ratio of about 1:2.^4–6^ Chordoid glioma is rare in children and rare outside the ventricle. Here, we report the case of a 10-year-old child with chordoid glioma in the right thalamic region. CT and MRI findings are not consistent with the typical third ventricular chordoid glioma and the chordate gliomas of the left thalamus reported by Kim et al..^7^

## Case report

A 10-year-old female child was hospitalized on 2 September 2015 with a paroxysmal headache that had persisted for 2 weeks. The physical examination on admission showed that the patient was lucid and demonstrated language fluency, but was otherwise uncooperative during the physical examination. There was no hoarseness, choking when drinking water or difficulty swallowing. There was no atrophy or hypertrophy of the overall body muscle, the muscle tension of the extremities was normal, and the muscle strength of the extremities was Grade 5. The bilateral finger nose and calcaneal knee tibia tests were stable and accurate. The bilateral Babinski sign was negative, the neck was soft and the Kirschner sign was negative.

**Figure 1.**
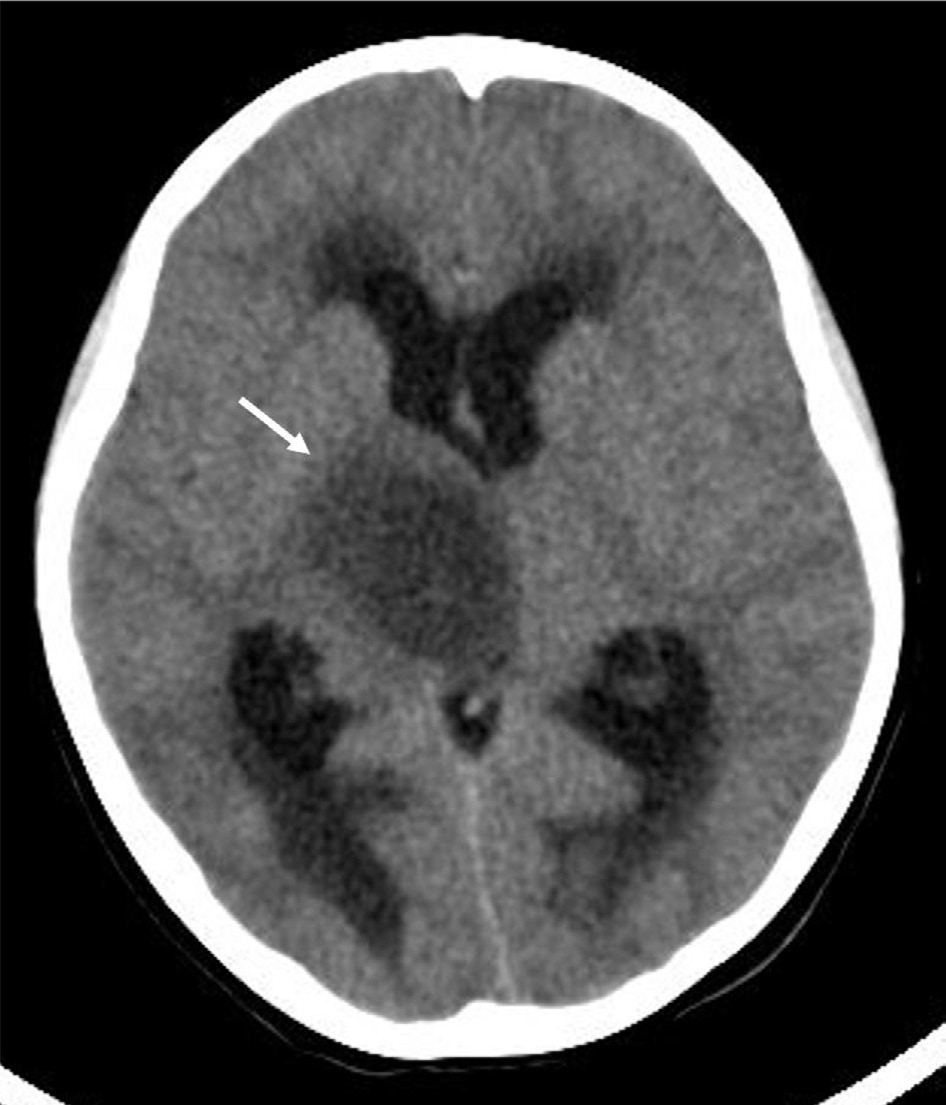
Computed tomography of the tumour (long white arrow). CT revealed an oval low-density mass in the right thalamus.

**Figure 2.**
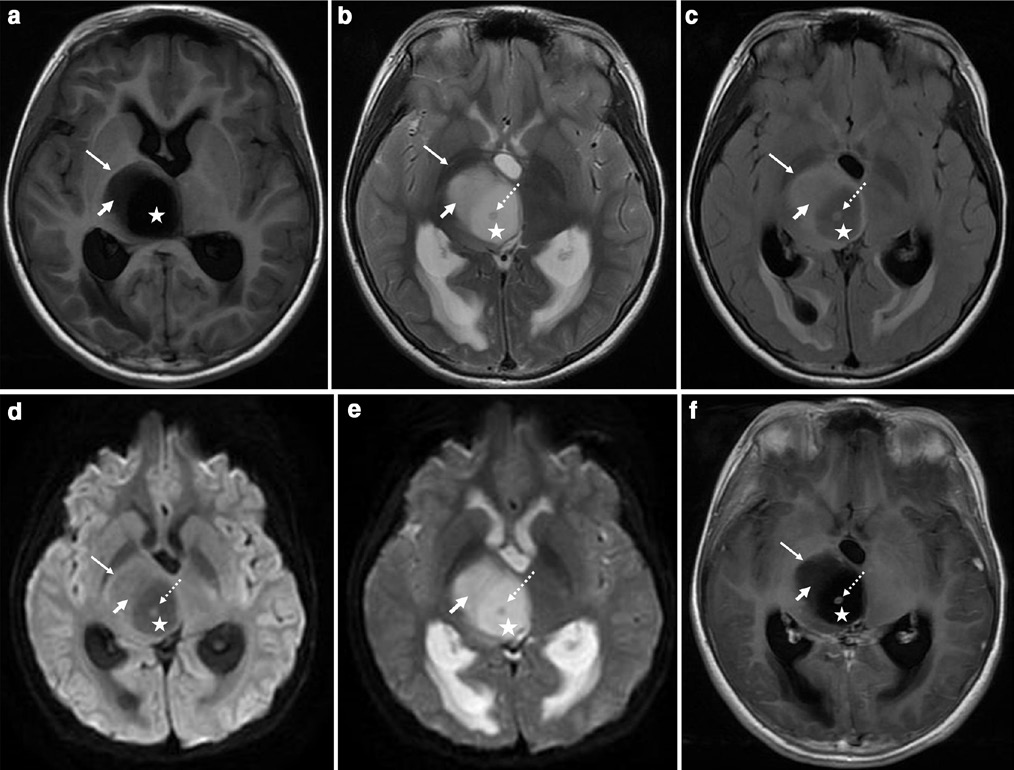
(A-F) Magnetic resonance images of the tumour (long white arrow). A and B, T1WI shows a well-defined solid cystic mass in the right thalamus. In the cystic part (pentagram), T1WI shows hypointensity, T2WI shows hyperintensity and slightly hyperintense nodules (white dotted arrow) are visible in the centre. The solid part (short white arrow) shows slight hypointensity in T1WI and slight hyperintensity in T2WI. C, Axial FLAIR shows slight hyperintensity in the cystic area and the central nodule, and the solid part shows hyperintensity. D and E, The lesion shows no diffusion restriction on diffusion-weighted imaging (DWI) and on the apparent diffusion coefficient map. F, Axial T1WI enhancement shows the annular enhancement of central nodule. (MRI = magnetic resonance imaging, T1WI = *T*_1_-weighted1-weighted images, and T2WI = *T*_2_-weighted2-weighted images).

CT revealed a low-density oval mass in the right thalamus (Figure 1). MRI examination showed an oval, solid cystic mass in the right thalamic region with clear boundaries; the cystic area (pentagram) was mostly in the centre of the lesion. T1W images showed hypointensity (Figure 2A), and T2W images showed hyperintensity (Figure 2B). Fluid-attenuated inversion recovery (FLAIR) showed slight hyperintensity (Figure 2C), and diffusion-weighted imaging (DWI) showed hypointensity (Figure 2D and E); the solid part (short white arrow) was mostly located at the periphery of the lesion. T1W images showed slight hypointensity (Figure 2A), T2W images and FLAIR showed slight hyperintensity (Figure 2B and C), and DWI showed isointensity (Figure 2D). There was a slightly hyperintense nodular signal visible on T2W images and on FLAIR in the central part of the cystic region (Figure 2B and C; white-dotted arrow), and DWI showed slight hyperintensity. The central nodule was enhanced with annular enhancement, but there was no readily visible enhancement in the cystic part and the surrounding solid part (Figure 2F). There was no readily visible oedema zone around the tumour, and the tumour compressed the third ventricle to cause obstructive hydrocephalus. Low-grade glioma was diagnosed before surgery.

**Figure 3.**
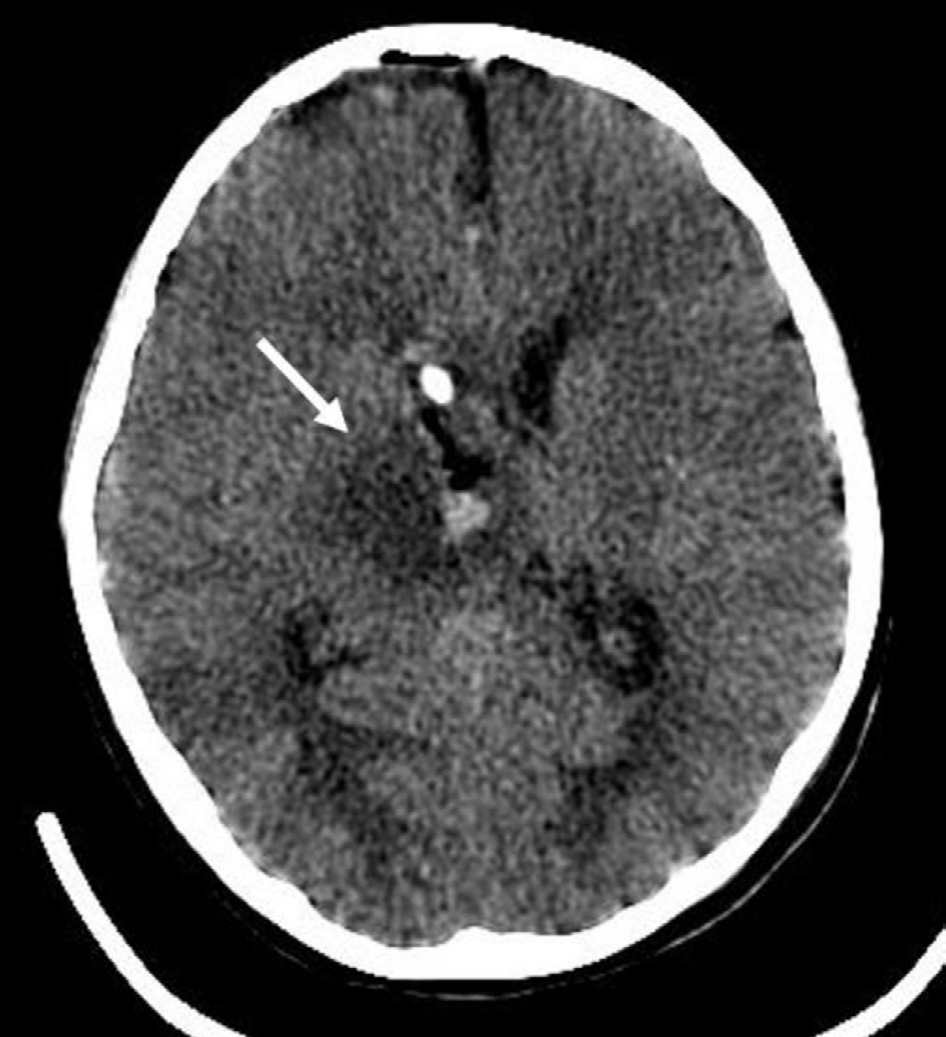
Postoperative computed tomography (CT) examination. The CT image shows that most of the tumours were resected and a little blood oozed in the operative area.

The patient underwent resection of the right thalamic space-occupying lesions via a transcortical, right lateral ventricle approach under general anesthesia on the morning of 8 September 2015. On the second day after surgery, CT showed that most of the tumour had been resected, and there was some bleeding in the surgical area (Figure 3).

**Figure 4.**
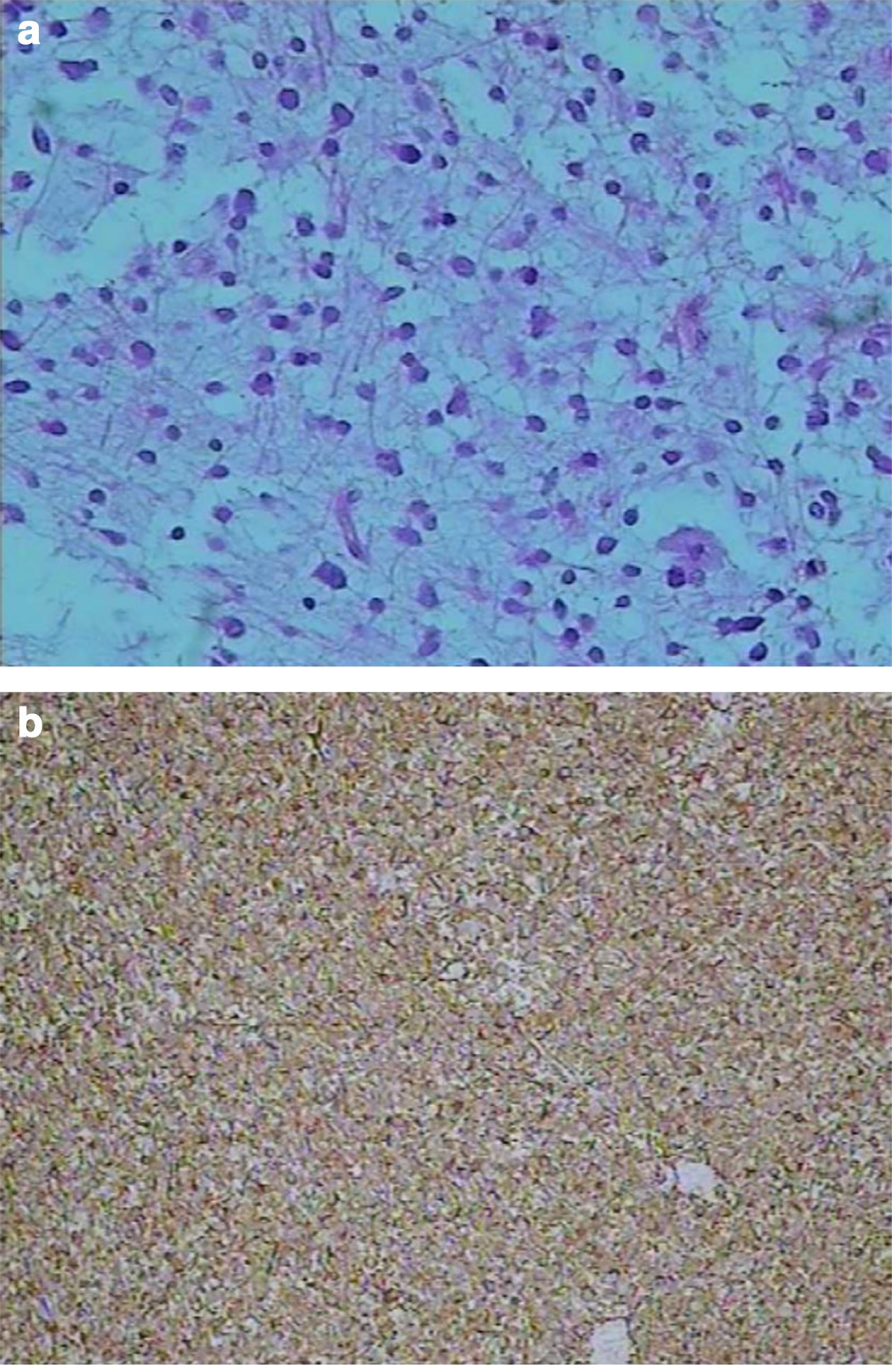
Histological examination of the chordoid glioma. A, Hematoxylin and eosin staining shows that the tumour cells were sparse, the arrangement was dense, the atypia was not obvious, there was no obvious proliferation of blood vessels, some of the tumour cells had degenerated, and there was interstitial myxoid degeneration (HE,×100). B, Immunohistochemical analyses of the tumour cells showing positive staining for GFAP (×40).

Postoperative pathology showed that the tumour cells were sparse, the arrangement was dense, the heterotype was not obvious, the blood vessels did not proliferate obviously, some of the tumour cells degenerated, and the interstitial myxoid was degenerated (Figure 4A). Immunohistochemically, glial fibrillary acidic protein (GFAP) (Figure 4B), synaptophysin (SYN), S-100 protein, neurofilament (NF), and Ki67 were all positive, and Neu-N was negative. Finally, the condition was diagnosed as chordoid glioma (WHO grade II).

The patient had recovered well by the 23rd day after the operation and mentioned no particular discomfort. Physical examination showed that the patient could speak clearly and cooperated with the physical examination. The head incision healed well. Pupil dilation occurred evenly across both eyes; the patient showed direct and indirect light reflex, and eye movement was normal. Muscle tension was normal, muscle strength in both limbs was Grade 5, and the bilateral pathological reflex was negative. The patient was discharged in a stable condition.

## Discussion

The origin of chordoid glioma is not clear. Brat et al,^8^ as well as studies by other researchers have reported that the tumour is located in the third ventricle and has ependymal differentiation and an ultrastructure similar to that of secretory ependymal cells, so it may be a subtype of ependymoma. Romero-Rojas et al^9^ suggested that the positive expression of CD99 indicates that a given tumour originated from ependymal cell differentiation, which indirectly confirms Brat et al.’s report. However, Sato et al^10^ found that the ultrastructure of chordoid gliomas shares greater similarity to that of embryonic elongated cells, which suggests that notochordal glioma may originate from elongated cells, but genes related to tumourigenesis have not been identified.

Typical chordoid gliomas are often solid masses that occupy the third ventricle, and a few of them may be complicated with cystic degeneration. The tumour signal is changeable. On MRI images, 63% of chordoid gliomas showed isointensity in T1W images, and the ratios of high signal, isointense signal, and low signal in T2W images were 42%, 37%, and 21%,^7^ respectively. The principal cause of signal variability was that the tumour was composed of epithelial-like cells arranged in clusters and stripes buried in the mucus-rich matrix, such that the number of clusters and stripes also changed the signal. When the epithelioid cells were dominant, the T2W images were mainly isointense and featured a low signal. When the mucus-like matrix was dominant, T2W images were visibly high signal. The main enhancement scan showed readily visible enhancement. The MRI findings of this case differed from those of typical chordoid gliomas. The main manifestations were cystic solid mass, cystic degeneration, cystic central solid nodules and enhanced nodules. Pathology showed that some of the tumour cells had undergone degeneration and interstitial myxoid degeneration, which was consistent with the observations made via MRI.

Chordoid glioma in the thalamus is different from pilocytic astrocytoma. Pilocytic astrocytoma usually occurs in adolescents and children, and it most commonly occurs in the cerebellar hemisphere. The main manifestations of MRI are large cysts and small nodules, and the nodules are located in the cyst wall.

The ideal treatment of choroid glioma is gross total resection (GTR) of the tumour, but because of its deep location, it is difficult to remove the tumour and the incidence of postoperative complications is very high, so subtotal resection (STR) is often chosen. It has been suggested that adjuvant γ knife radiosurgery (GKRS) following planned STR may be a safe, effective and minimally invasive strategy for choroid glioma in patients at high risk for the GTR of tumours.^11^

## Conclusion

We reported a case of chordoid glioma in a rare location in a child. The patient’s imaging findings differed from those of a typical chordoid glioma.

Highlights:

1. Chordoid glioma is a rare intracranial tumour, which usually occurs in middle-aged female patients – mainly in the third ventricle, hypothalamus and suprasellar region.
2. The reason for the observed signal variability is primarily due to the fact that the tumour is composed of epithelial-like cells arranged in clusters and stripes buried in the mucus-rich matrix; therefore, the number of the two components also leads to signal changes.
3. Through the study of this case, we recognized the atypical imaging manifestations of a chordoid glioma in a rare location.
